# Clinical outcomes of surgical resection for recurrent lesion after curative esophagectomy for esophageal squamous cell carcinoma: a nationwide, large-scale retrospective study

**DOI:** 10.1007/s10388-021-00878-2

**Published:** 2021-09-12

**Authors:** Kensuke Kudou, Hiroshi Saeki, Yuichiro Nakashima, Yasue Kimura, Eiji Oki, Masaki Mori, Mototsugu Shimokawa, Yoshihiro Kakeji, Yasushi Toh, Yuichiro Doki, Hisahiro Matsubara

**Affiliations:** 1 Department of Surgery and Science, Graduate School of Medical Sciences, Kyushu University, Fukuoka, Japan; 2 Department of Gastroenterological Surgery, Clinical Research Institute Cancer Research Division, National Hospital Organization Kyushu Medical Center, Fukuoka, Japan; 3 Department of General Surgical Science, Gastroenterological Surgery, Graduate School of Medicine, Gunma University, 3-39-22 Showa-machi, Maebashi, Gunma 371-8511 Japan; 4 School of Medicine, Tokai University, Tokyo, Japan; 5 Department of Biostatistics, Yamaguchi University Graduate School of Medicine, Yamaguchi, Japan; 6 Clinical Research Institute, National Hospital Organization Kyushu Cancer Center, Fukuoka, Japan; 7 Division of Gastrointestinal Surgery, Department of Surgery, Graduate School of Medicine, Kobe University, Kobe, Japan; 8 Department of Gastroenterological Surgery, National Hospital Organization Kyushu Cancer Center, Fukuoka, Japan; 9 Department of Gastroenterological Surgery, Graduate School of Medicine, Osaka University, Suita, Osaka Japan; 10 Department of Frontier Surgery, Graduate School of Medicine, Chiba University, Chiba, Japan

**Keywords:** Esophageal squamous cell carcinoma, Esophagectomy, Recurrence

## Abstract

**Background:**

Several studies have reported the efficacy of resection for recurrent lesions. However, they involved a limited number of subjects. This study aimed to identify a subset of patients who benefit from surgical resection of recurrent lesions after curative esophagectomy for esophageal squamous cell carcinoma.

**Methods:**

Clinicopathological features of 186 patients with esophageal squamous cell carcinoma who underwent surgical treatment for postoperative recurrent lesions at 37 accredited institutions of the Japanese Esophageal Society were evaluated.

**Results:**

The most common recurrence site was the lymph node (106 cases; 58.6%), followed by the lung (40 cases; 22.1%). Univariate analyses revealed that pN 0–1 at esophagectomy (*P* = 0.0348), recurrence-free interval of ≥ 550 days (*P* = 0.0306), R0 resection (*P* < 0.0001), and absence of severe complications after resection for recurrent lesions (Clavien–Dindo grade < IIIa) (*P* = 0.0472) were associated with better overall survival after surgical resection. According to multivariate analyses, pN 0–1 (*P* = 0.0146), lung metastasis (*P* = 0.0274), recurrence-free interval after curative esophagectomy of ≥ 550 days (*P* = 0.0266), R0 resection (*P* = 0.0009), and absence of severe complications after resection for recurrent lesions (Clavien–Dindo grade < IIIa) (*P* = 0.0420) were independent predictive factors for better overall survival.

**Conclusions:**

Surgical resection of recurrent esophageal squamous cell carcinoma lesions is a useful option, especially for cases involving lower pN stage, lung metastasis, long recurrence-free intervals after esophagectomy, and technically resectable lesions. Surgical risks should be minimized as much as possible.

**Supplementary Information:**

The online version contains supplementary material available at 10.1007/s10388-021-00878-2.

## Introduction

Recurrence after radical surgery (R0 resection) for esophageal cancer occurs in 28–47% of patients in Japan [1], and several studies performed in Western countries have reported recurrence rates of > 50% [2–4]. The median time to recurrence was 10–12 months, and the median survival time was 7–18 months [4, 5]. A previous study reported that 90% of recurrent esophageal cancer cases occurred within 38 months after surgery, and that half of those cases recurred within 12 months [4]. Long-term survival and complete cure, especially for the subset of patients who underwent successful surgical resection for lymph node (LN) or lung recurrence, have been reported; therefore, aggressive treatment for recurrent diseases could be considered for some patients [4–11].

The significance of local treatment for recurrent esophageal cancer has not yet been ascertained. When recurrence occurs in localized or resectable regions after radical esophagectomy, surgical resection, radiotherapy, and chemoradiotherapy are considered effective treatments [12]. Recurrence is frequently observed in the LNs, lungs, liver, and bone [13]. Other studies reported that anastomotic LNs, supraclavicular LNs, and mediastinal LNs were the most common local recurrence sites [4, 14, 15]. Several studies have demonstrated the efficacy of lymphadenectomy for localized LN recurrence, such as cervical LNs, but these studies were performed at a single center or involved only a limited number of cases [6–10]. Regarding resection for local recurrence and recurrent lesions of other organs, such as the lung, liver, brain, parotid gland, and pancreas, only reports based on a small number of cases are available; therefore, its efficacy is still undetermined [11, 16–18]. Su et al. reported that the time of recurrence after curative esophagectomy (< 12 months), pattern of recurrence (local–regional recurrence defined as anastomotic recurrence and/or occurring in the mediastinum, upper abdomen, or cervical area), and treatment of recurrence (radiotherapy, surgery, or chemotherapy) were independent favorable prognostic factors for patients with recurrence after complete resection of esophageal carcinoma [13]. Of note, during this study, treatments for recurrent lesions included a mixture of modalities such as radiotherapy, chemotherapy, chemoradiotherapy, and radiofrequency ablation, and the proportion of patients who underwent surgical resection was approximately 5% [13]. Therefore, the clinical significance of surgical resection for recurrence in patients with esophageal carcinoma remains controversial. It is important to investigate the indications for surgical resection and the efficacy of surgical resection for recurrence, and to identify subgroups in which a favorable long-term prognosis can be expected after resecting the recurrent lesions.

We conducted a nationwide survey of patients with recurrent esophageal squamous cell carcinoma (ESCC) treated at institutions accredited by the Japan Esophageal Society. We focused on ESCC, which accounts for ~ 90% of the histological types in Japan, to avoid confusion with different types of carcinomas. This study aimed to clarify the current state of resection of recurrent lesions and to identify a subset of patients who can be expected to have a favorable long-term prognosis. To our knowledge, this is the largest study of surgery for recurrent ESCC to date, and we believe that the results will be of great significance to practical treatment strategies.

## Methods

### Study design and patients

This study was approved as a research project by the Japanese Esophageal Society in 2017. We conducted a questionnaire-based, retrospective clinical review of patients with recurrent ESCC who underwent surgical treatment for recurrent lesions after curative esophagectomy at institutes accredited by the Japan Esophageal Society between January 2009 and December 2013. Of the 105 facilities, we received responses from 58; 21 of these 58 facilities had no applicable cases. Finally, the data from 37 facilities were analyzed during the present study. The questionnaire was prepared at Kyushu University, approved by the ethics review committee, and then sent to the participating facilities of the Japan Esophageal Society. Characteristics such as sex, age, tumor location, clinical stage, pathological stage, comorbidities, surgical procedure, pathological effect of neoadjuvant therapy, postoperative complications, details of perioperative therapy, number of recurrent lesions, recurrence site, residual tumor, recurrence-free interval, and long-term prognosis were investigated using this questionnaire. Cancer staging was based on the TNM classification. The clinical and pathological data of 186 patients with histologically diagnosed ESCC who underwent surgical treatment for postoperative recurrent lesions were collected from the participating institutions. Patients with adenocarcinoma or other tumors were excluded from the study. Permission to perform this retrospective cohort study was provided by the Institutional Review Board of Kyushu University (29–325) and the other 36 institutions.

### Statistical analyses

Survival curves were plotted according to the Kaplan–Meier method, and differences were analyzed using the log-rank test and a Cox proportional hazard model. Overall survival (OS) during this study was measured from the date of surgical resection for recurrent lesions. Univariate and multivariate analyses were performed using a Cox proportional hazards model to identify independent prognostic factors. All *P *values were two-sided, and *P* < 0.05 was considered statistically significant. A receiver-operating characteristic curve analysis was used to identify optimal cut-off values. All analyses were performed using JMP PRO 11 software (SAS Institute Inc., Cary, NC; https://www.jmp.com/ja_jp/home.html).

## Results

### Patient characteristics at the time of initial esophagectomy for primary ESCC

The clinicopathological characteristics of 186 ESCC patients (159 [85.5%] men and 27 [14.5%] women) are summarized in Table 1. The median patient age at the time of primary esophagectomy was 63.3 years (range, 40–83 years). The middle thorax was the most frequent (48.4%) tumor location, followed by the lower thorax (25.3%), upper thorax (16.7%), cervix (4.8%), and abdomen (4.8%). Regarding the surgical procedure for the primary lesion, 167 (89.8%) patients underwent subtotal esophagectomy and reconstruction. Postoperative complications occurred in 93 (50.0%) patients after surgery for the primary lesion. The degree of postoperative complications was categorized according to the Clavien–Dindo classification [19, 20]. Severe complications (Clavien–Dindo grade ≥ IIIa) occurred in 19.9%. Major complications included recurrent nerve paralysis (13.4%), respiratory complications (12.9%), and anastomotic leakage (10.8%). Details of neoadjuvant and adjuvant therapies associated with esophagectomy are summarized in Online Resource 1.

**Table 1:** Clinicopathological characteristics of patients at initial esophagectomy

Characteristic	No. of patients	
	*n*	(%)
*Sex*		
Male	159	(85.5)
Female	27	(14.5)
*Age*		
63.3		(40–83)
*Tumor location*		
Cervix	9	(4.8)
Upper thorax	31	(16.7)
Middle thorax	90	(48.4)
Lower thorax	47	(25.3)
Abdomen	9	(4.8)
*cStage*		
I	23	(12.4)
II	61	(32.8)
III	88	(47.3)
IVa	14	(7.5)
*cT*		
T1	43	(23.1)
T2	38	(20.4)
T3	92	(49.5)
T4	13	(7.0)
*cN*		
N0	53	(28.5)
N1	57	(30.6)
N2	47	(25.3)
N3	19	(10.2)
N4	10	(5.4)
*Comorbidities and previous history*		
No	114	(61.3)
Cardiovascular disease	17	(9.1)
Hypertension	17	(9.1)
Respiratory disease	13	(7.0)
Malignant tumor	11	(5.9)
Diabetes	10	(5.4)
Liver disease	7	(3.8)
Cerebrovascular disease	6	(3.2)
Others	9	(4.8)
*Surgical procedure*		
Subtotal esophagectomy	167	(89.8)
Laryngopharyngoesophagectomy	7	(3.8)
Others	12	(6.5)
*All postoperative complications*		
No	93	(50.0)
Yes	93	(50.0)
*Severe complications (CD grade ≥ IIIa)*		
No	149	(80.1)
Yes	37	(19.9)
*Details of complications*		
No	93	(50.0)
Recurrent nerve paralysis	25	(13.4)
Respiratory complication	24	(12.9)
Anastomotic leakage	20	(10.8)
Surgical site infection	15	(8.1)
Cardiovascular complication	12	(6.5)
Chylothorax	9	(4.8)
Bleeding	4	(2.2)
Others	16	(8.6)
*pStage*		
0	12	(6.5)
I	26	(14.0)
II	55	(29.6)
III	81	(43.5)
IVa	12	(6.5)
*pT*		
T0	13	(7.0)
T1	58	(31.2)
T2	22	(11.8)
T3	84	(45.2)
T4	9	(4.8)
*pN*		
N0	64	(34.4)
N1	45	(24.2)
N2	50	(26.9)
N3	21	(11.3)
N4	6	(3.2)
*Pathological effect of neoadjuvant therapy* (*n *= 111)		
Grade 0	13	(7.0)
Grade 1a	51	(27.4)
Grade 1b	12	(6.5)
Grade 2	16	(8.6)
Grade 3	12	(6.5)
Unknown	7	(3.8)

Data are presented as *n* (%), with the exception of age, which is presented as mean (range)

*CD* Clavien–Dindo classification

### Clinical features at the time of postoperative recurrence

**Fig. 1:**
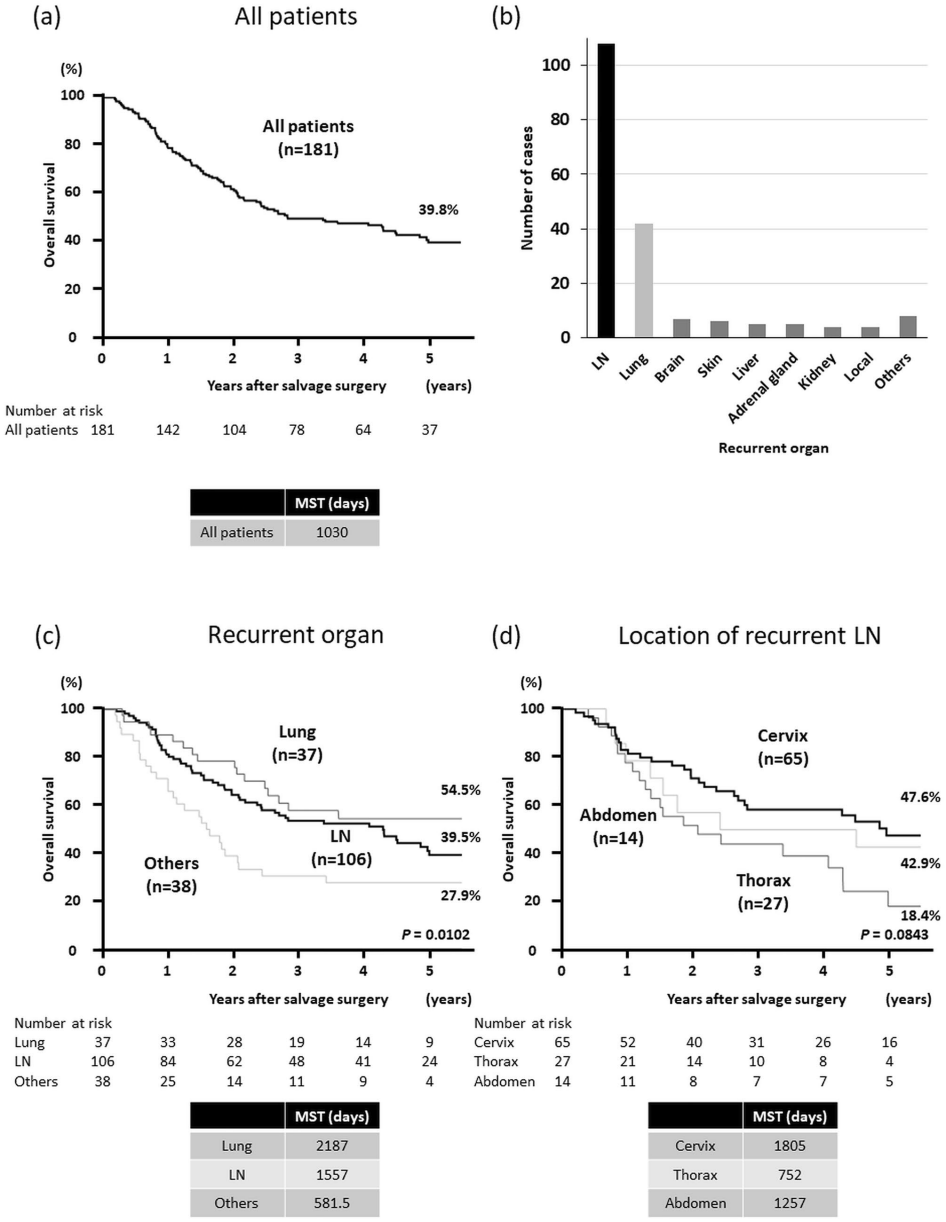
**a** Overall survival (OS) after recurrence for 181 patients. The median survival time after surgical resection for recurrent lesions was 1030 days and the 5-year OS rate was 39.8%. **b** Categorization by organs and number of cases. **c** Kaplan–Meier curves of OS after surgical resection for recurrent lesions among three groups created based on the organs where recurrence was observed (lymph nodes, lung, and other organs). **d** Kaplan–Meier curves of OS after surgical resection for recurrent lesions among patients with lymph-node metastases categorized into three groups created according to the location of recurrent lesions (cervix, thorax, and abdomen). *LN* lymph node, *MST* median survival time

The clinical features related to surgery for recurrent lesions are summarized in Table 2. The median interval from the date of prior esophagectomy to the date of recurrence was 351 days (range, 57–3556 days). Regarding the number of recurrent lesions targeted for resection, 148 cases (79.6%) involved solitary lesions in a single organ, 24 (12.9%) involved multiple lesions in a single organ, and 14 (7.5%) involved multiple lesions in multiple organs. Among the 186 patients, 157 (84.4%) underwent complete resection (R0 resection), 12 (6.5%) had microscopic residual disease (R1 resection), and 17 (9.1%) had macroscopic residual disease (R2 resection). The incidence of postoperative complications for recurrent lesions was lower than that after surgery for primary esophagectomy (all grades: 10.2% vs. 50.0%; severe [Clavien–Dindo grade ≥ IIIa]: 3.8% vs. 19.9%). Severe complications after surgery for recurrent lesions included respiratory complications, bleeding, recurrent nerve paralysis, pyothorax, and necrosis of the reconstructed intestine. Recurrence most frequently occurred in the LN (108 cases; 58.1%), followed by the lung (42 cases; 22.6%), brain (7 cases; 3.8%), skin (6 cases; 3.2%), and liver and adrenal glands (5 cases; 2.7%) (Fig. 1b).

**Table 2:** Clinical features related to surgery for recurrent lesion

Characteristic	No. of patients	
	*n*	(%)
*Number of recurrent lesions*		
Solitary	148	(79.6)
Multiple	38	(20.4)
*Number of organs with recurrence*		
Single	172	(92.5)
Multiple	14	(7.5)
*Residual tumor*		
R0	157	(84.4)
R1	12	(6.5)
R2	17	(9.1)
*All postoperative complications*		
No	167	(89.8)
Yes	19	(10.2)
*Severe complications (CD grade ≥ IIIa)*		
No	179	(96.2)
Yes	7	(3.8)
*Details of complications*		
No	167	(89.8)
Recurrent nerve paralysis	5	(2.7)
Respiratory complication	3	(1.6)
Bleeding	3	(1.6)
Surgical site infection	3	(1.6)
Others	5	(2.7)

*CD* Clavien–Dindo classification

The details of therapy before and after surgical resection for recurrent lesions were also investigated. Twenty-five percent of patients underwent chemotherapy and/or radiotherapy before surgical resection for recurrent lesions. Therapy after surgery for recurrent lesions was performed for 45% of patients. Details about chemotherapy regimens and radiation exposure doses are shown in Online Resource 2.

### Survival based on recurrence location

Among the 186 ESCC patients, 5 were excluded from the prognosis analyses during this study because of the lack of available data regarding survival time. Therefore, for 181 patients, the median survival time after surgery for recurrent lesions was 1030 days (range, 64–2748 days) and the 5-year OS rate was 39.8% (Fig. 1a). Patients were divided into three groups according to the recurrence location (LN, lung, or other organs), and the Kaplan–Meier method was used to compare the OS after surgery for recurrent lesions. The 5-year OS rates were better for patients who underwent surgical resection of the LN or lung than for those who underwent surgical resection of other organs (LN, 39.5%; lung, 54.5%; other organs, 27.9%; *P* = 0.0102) (Fig. 1c). Between-group comparisons indicated that the prognosis for patients whose target lesions were in the LN or lung was significantly better than that for patients with target lesions in other organs (hazard ratio [HR]: LN vs. others, 0.555, *P* = 0.0159; HR: lung vs. others, 0.440, *P* = 0.0065; HR: lung vs. LN, 0.792, *P* = 0.3771).

Subgroup analyses were performed. The Kaplan–Meier method was performed for subjects with LN metastases by categorizing them into three groups according to the recurrent lesion location (cervix, thorax, and abdomen). Patients with thoracic LN metastases had a relatively poor prognosis compared to patients with recurrence in the other two locations (*P* = 0.0843) (Fig. 1d). Comparisons of each group indicated that the prognosis for the thorax group was significantly poorer than that for the cervix group (HR: thorax vs. cervix, 1.865, *P* = 0.0354; HR: thorax vs. abdomen, 1.661, *P* = 0.2132; HR: abdomen vs. cervix, 1.123, *P* = 0.7736).

### Survival after recurrent lesion resection

**Fig. 2:**
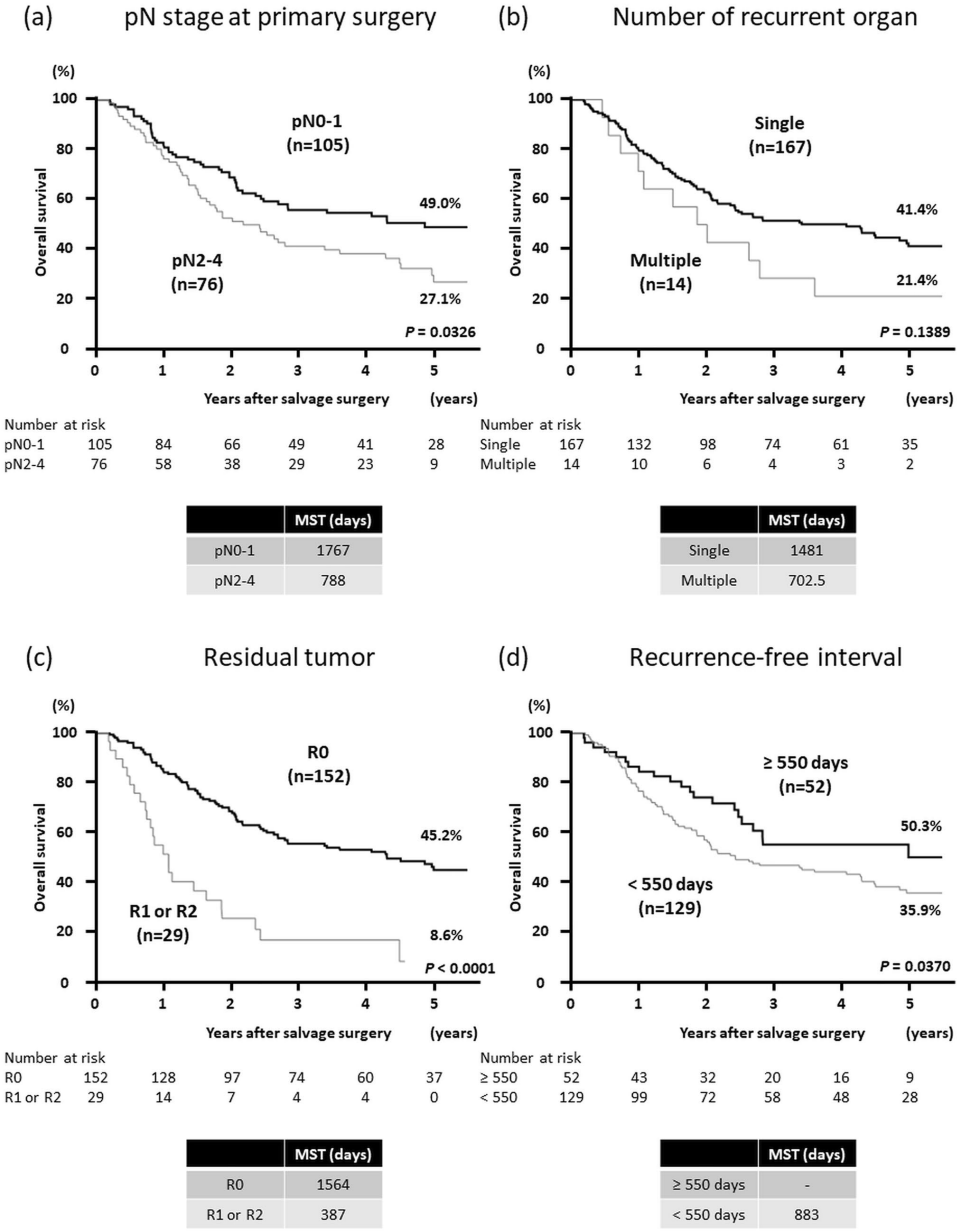
Kaplan–Meier curves of overall survival after surgical resection for recurrent lesions for **a** patients with pN0-1 and pN2-4 at primary esophagectomy, **b** patients who underwent resection and had single-organ metastases and multiple-organ metastases, and **c** patients who underwent radical surgery (R0) and non-curative resection (R1 or R2). **d** Kaplan–Meier curves of overall survival based on the length of the recurrence-free interval after primary esophagectomy. The cut-off value for recurrence-free interval was 550 days, which was obtained by the receiver-operating characteristic curve analysis. *R0* radical resection, *R1* microscopic residual disease, *R2* macroscopic residual disease, *MST* median survival time

The Kaplan–Meier method was performed by categorizing the patients into two groups according to the pN stage at primary esophagectomy (pN0-1 vs. pN2-4), the number of resected recurrent organs (single organ vs. multiple organs), or whether complete resection for recurrent lesions was achieved (R0 surgery vs. R1 or R2 surgery). The results revealed that pN0 and pN1 (HR, 0.659; *P* = 0.0326) and R0 surgery (HR, 0.299; *P* < 0.0001) were significantly correlated with better prognoses (Fig. 2a, c), and that single-organ metastasis (HR, 0.626; *P* = 0.1389) tended to be correlated with better OS, but not significantly (Fig. 2b).

We analyzed the relationship between the length of the recurrence-free interval after primary esophagectomy and prognosis after recurrence. The cut-off value was identified as 550 days by the receiver-operating characteristic curve analysis. Patients whose recurrence-free interval was ≥ 550 days had a better prognosis after resection of the recurrent lesion (HR, 0.604; *P* = 0.0370) (Fig. 2d).

### Survival according to the development of complications after recurrent lesion resection

**Fig. 3:**
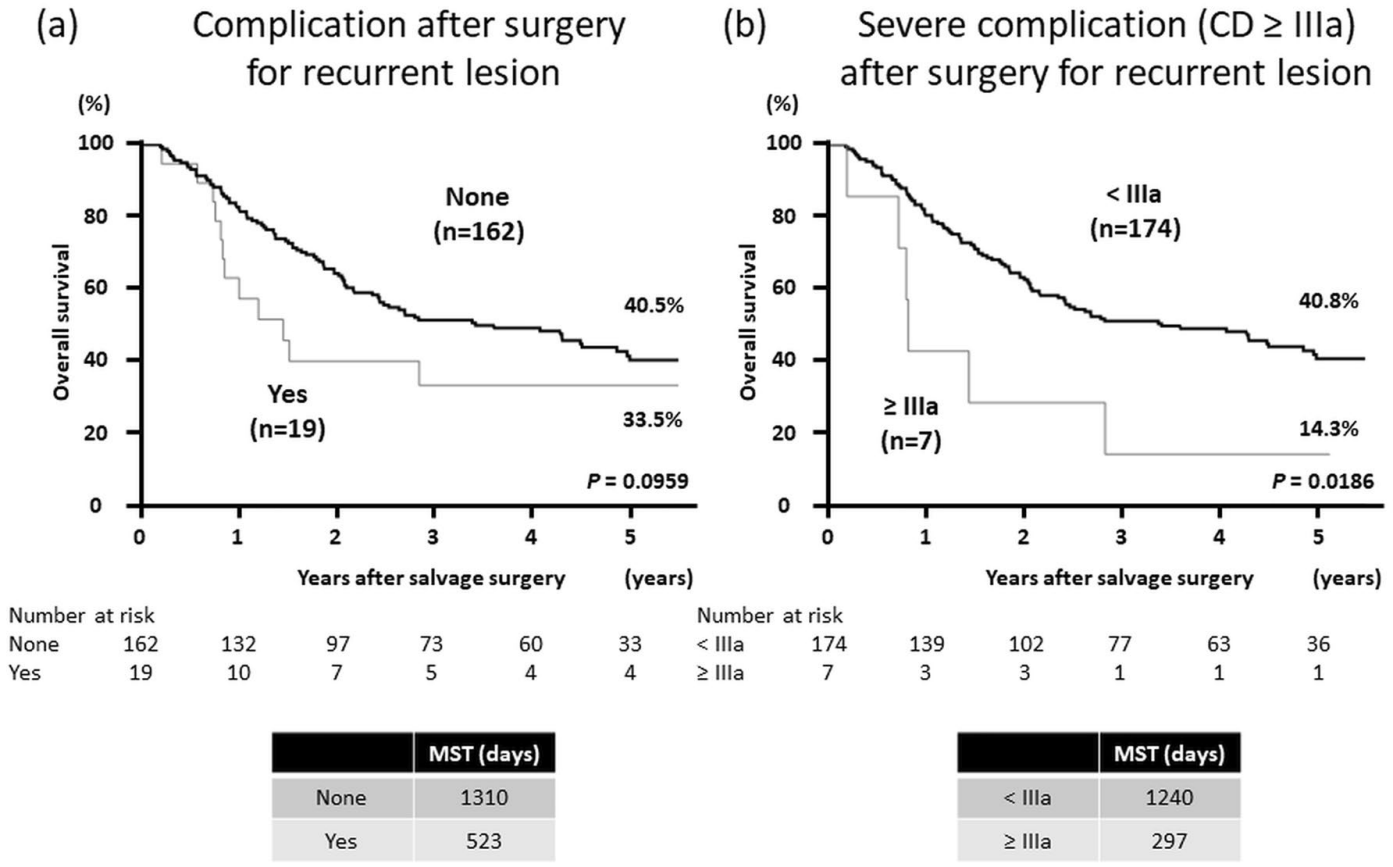
Kaplan–Meier curves of overall survival after surgical resection for recurrent lesions in patients with and without complications after surgical resection for recurrent lesions. **a** Patients with and without complications and all Clavien–Dindo grades. **b** Patients with and without severe complications (Clavien–Dindo grade ≥ IIIa). *MST* median survival time, *CD* Clavien–Dindo grade

We also analyzed the correlation between the prognosis and the occurrence of complications after surgical resection for recurrent lesions. The OS rate of patients with complications after surgical resection for recurrent lesions tended to be poorer than that of patients without complications (*P* = 0.0959) (Fig. 3a). The OS rate of those with severe complications (Clavien–Dindo grade ≥ IIIa) was significantly poorer than that of patients without severe complications (*P* = 0.0186) (Fig. 3b).

### Efficacy of therapy before and after surgical resection for recurrent lesions

**Fig. 4:**
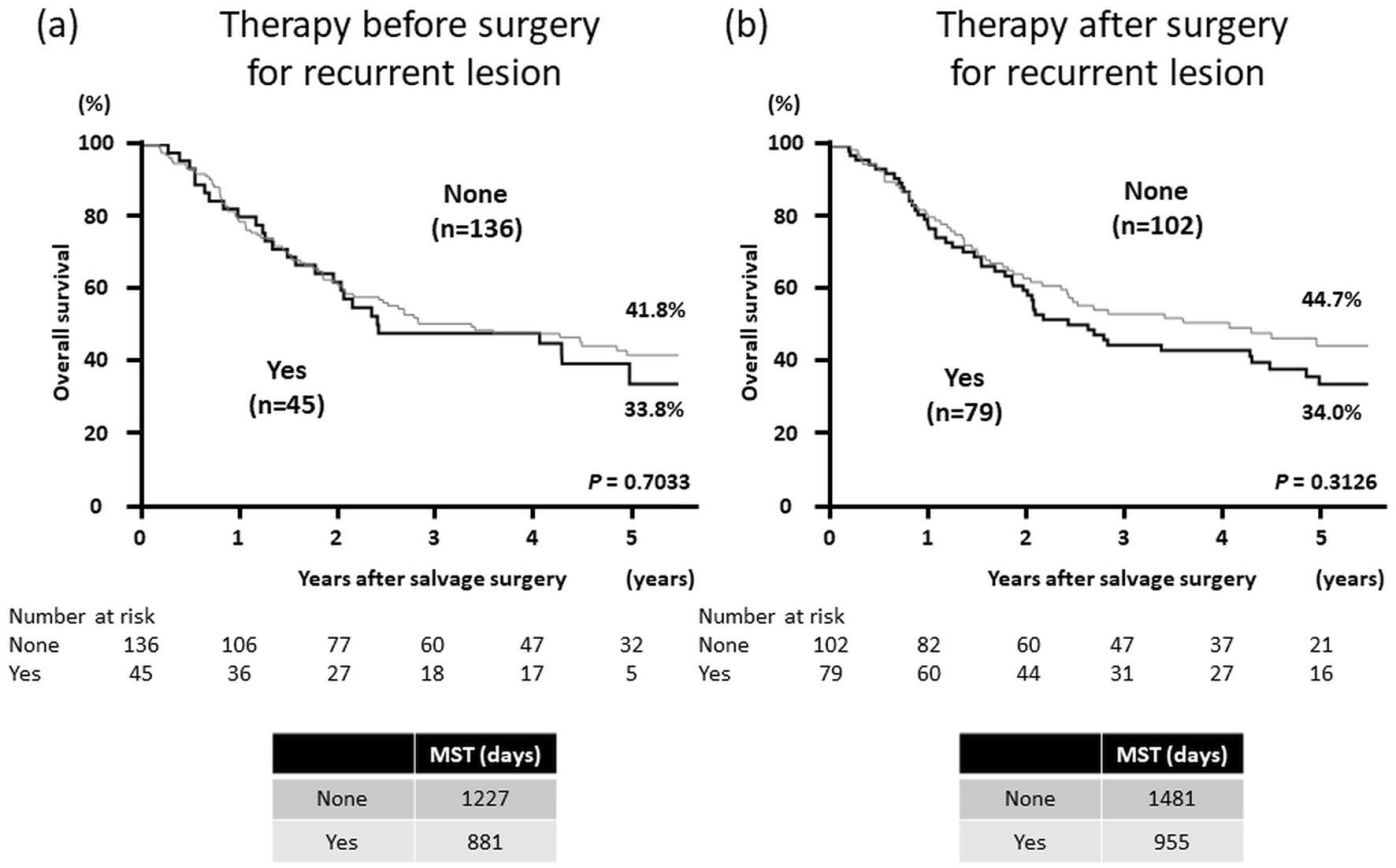
Kaplan–Meier curves of overall survival after surgical resection for recurrent lesions according to treatment with chemotherapy and/or radiotherapy **a** before and **b** after surgical resection for recurrent lesions. *MST* median survival time

To verify the efficacy of perioperative chemotherapy and/or radiotherapy after recurrence, patients were divided into two groups according to the use of chemotherapy and/or radiotherapy before or after surgical resection for recurrent lesions and their OS rates were compared. The Kaplan–Meier curves showed that perioperative chemotherapy and/or radiotherapy did not improve OS after surgical resection for recurrent lesions (Fig. 4). The clinicopathological characteristics of patients according to treatment with chemotherapy and/or radiotherapy before and after surgical resection for recurrent lesions are summarized in Online Resources 3–6. There were no significant differences in clinical features between the two groups categorized according to the treatment before surgery for recurrent lesions (Online Resources 3 and 4). On the other hand, the rates of R0 resection (*P* = 0.0069) and single-organ recurrence (*P* = 0.0095) were significantly lower in patients who underwent treatment after surgery for recurrent lesions than in those who did not undergo postoperative treatment (Online Resources 5 and 6). Subgroup analyses were also performed according to the number of recurrent lesions and organs or the location of recurrent lesions, and no significant difference in the OS rates was observed (Online Resources 7–9).

### Factors associated with favorable prognoses after recurrent lesion resection

To identify independent prognostic factors for favorable OS after recurrent lesion resection, univariate and multivariate analyses with a Cox proportional hazards model were performed. If the *P *value of a factor was < 0.1 in the univariate analyses, then we included that factor in the multivariate analyses. Univariate analyses revealed that pN0 and pN1 at esophagectomy (*P* = 0.0348), recurrence-free interval of ≥ 550 days (*P* = 0.0306), R0 resection (*P* < 0.0001), and absence of severe complications after resection for recurrent lesions (Clavien–Dindo grade < IIIa) (*P* = 0.0472) were associated with better OS after surgical resection for recurrent lesions. We also performed univariate analyses using pN0 as the control. HR was 0.779 for pN0 vs pN1–4 (*P* = 0.2392) and 0.669 for pN0 vs pN2–4 (*P* = 0.0747). In the multivariate analyses, pN0 and pN1 (HR, 0.594; *P* = 0.0146), lung metastasis (HR, 0.529; *P* = 0.0274), recurrence-free interval of ≥ 550 days (HR, 0.584; *P* = 0.0266), R0 resection (HR, 0.389; *P* = 0.0009), and absence of severe complications after recurrent lesion resection (Clavien–Dindo grade < IIIa) (HR, 0.352; *P* = 0.0420) were independent predictive factors for better OS for patients who underwent surgical resection of recurrent lesions (Table 3).

**Table 3:** Univariate and multivariate analyses for overall survival

Factor	Univariate analysis		Multivariate analysis	
	HR (95% CI)	*P* value	HR (95% CI)	*P* value
Female (vs male)	0.744 (0.396–1.284)	0.3027	0.789 (0.416–1.383)	0.4245
Age ≥ 65 y (vs < 65 y)	0.975 (0.659–1.433)	0.8962	0.972 (0.655–1.437)	0.8889
pT 0–2 (vs pT 3–4)	0.764 (0.517–1.125)	0.1733	1.115 (0.720–1.725)	0.6249
pN 0–1 (vs pN 2–4)	0.659 (0.448–0.970)	0.0348	0.594 (0.391–0.902)	0.0146
LN (vs other organs)	0.840 (0.571–1.245)	0.3819	0.655 (0.405–1.066)	0.0881
Lung (vs other organs)	0.647 (0.386–1.033)	0.0687	0.529 (0.292–0.932)	0.0274
Solitary lesion (vs multiple)	0.701 (0.453–1.122)	0.1346	0.711 (0.408–1.332)	0.2723
Single organ (vs multiple)	0.626 (0.350–1.241)	0.1678	1.220 (0.511–2.983)	0.6543
Recurrence-free interval ≥ 550 days	0.604 (0.365–0.956)	0.0306	0.584 (0.348–0.941)	0.0266
R0 resection (vs R1 or R2)	0.299 (0.190–0.487)	< 0.0001	0.389 (0.234–0.669)	0.0009
PC < IIIa (surgery for recurrence)	0.384 (0.183–0.987)	0.0472	0.352 (0.153–0.959)	0.0420

*PC* Postoperative complication after surgical resection for recurrent lesion (Clavien–Dindo classification)

### R0 resection rate by each site of recurrence

The results of univariate and multivariate analyses indicated that R0 resection showed a strong correlation with better OS after surgical resection of recurrent lesions (Table 3). On the basis of this finding, we investigated the R0 resection rate by each site of recurrence. With regard to LN metastases according to the site of the recurrent lesion (cervix, thorax, and abdomen), the R0 resection rate was lower for thoracic LN metastases than for cervical and abdominal LN metastases. With regard to other organs, the R0 resection rate was higher for the lungs (95%) and liver (100%) than for other organs (Online Resource 10).

## Discussion

When recurrence occurs in a localized region or regions after radical surgery for esophageal cancer, surgery, chemoradiotherapy, and radiotherapy are considered treatments that can lead to radical cure [12]. Several studies that evaluated the efficacy of surgical resection for recurrent lesions after radical esophagectomy have suggested that resection of the LN or lung metastases yields a survival benefit for properly selected patients, but the benefit of resection for hepatic metastases remains controversial [7, 9, 11, 18]. A few studies indicated that successful resection for metastases of other organs, such as the brain, parotid gland, or pancreas, or for local recurrence may improve prognosis, although these results were based on one or a few cases [16, 17]. Many studies have highlighted the usefulness of chemoradiotherapy and radiotherapy for localized recurrence after radical surgery [21–24], and they are widely used in actual clinical practice. However, Ma et al. retrospectively compared the long-term outcomes of lymphadenectomy for cervical LN recurrence compared with those of chemoradiotherapy/radiotherapy and reported that patients who underwent lymphadenectomy had a better prognosis than those who received chemoradiotherapy/radiotherapy [10]. Most previous studies (including our study) demonstrated that the 5-year survival rates were 3–13% for patients who underwent chemoradiotherapy/radiotherapy for recurrent esophageal cancer [10, 21–24], and that their outcomes were poorer than those of patients who underwent surgical resection for recurrent lesions. However, no prospective comparative study has evaluated a suitable treatment strategy for postoperative recurrent ESCC.

Some small cohort studies have suggested that surgical resection might be useful, especially for localized recurrence in the LNs and lung in selected patients. However, the characteristics of ESCC patients who would benefit from recurrent lesion resection have not been ascertained. Therefore, it is necessary to identify the subset of patients who would benefit from surgical resection and identify the indications for surgery. Previous studies have reported that a higher primary N stage, shorter disease-free survival after primary esophagectomy, and multiple recurrences in cervical LNs were associated with poor prognoses after salvage treatment [25–27]. Moreover, Wang et al. analyzed 66 cases of salvage lymphadenectomy for ESCC recurrence in the cervical LNs and observed that a lower primary N stage and R0 resection of recurrent nodes were favorable prognostic factors [9]. During the present study, we investigated the outcomes of surgical resection of various organ metastases in a large cohort. Our results suggest that lower pN stage, lung metastasis, long recurrence-free interval after esophagectomy, and technically resectable lesions are favorable prognostic factors for OS after resection of recurrent lesions in ESCC patients; these results are consistent with those of previous studies. In particular, R0 resection showed a strong correlation with better OS after surgical resection of recurrent lesions. Accordingly, we compared the R0 resection rate by each site of recurrence (Online Resource 10) and found a lower rate for thoracic LN metastases than for cervical and abdominal LN metastases. This result may be correlated with the worse prognosis of thoracic LN metastases (Fig. 1d).

There are concerns that surgical complications could reduce long-term survival. It has been shown that the survival rates of patients who developed postoperative complications after curative resection for primary ESCC were significantly poorer than those of patients who did not [28, 29]. To date, no studies of ESCC have addressed the question of whether postoperative complications after resection for recurrent lesions have an impact on long-term survival. During the present study, severe complications after surgical resection for recurrent lesions were correlated with a poor prognosis (Fig. 3b). During the multivariate analysis, the occurrence of severe complications (Clavien–Dindo grade ≥ IIIa) after resection for recurrent lesions was identified as an independent prognostic factor for poor OS (*P* = 0.0420) (Table 3). These results suggest that excessively invasive surgery should be avoided through appropriate evaluation of the anatomical features of the recurrent site and the general condition of the patient. However, further studies are necessary to confirm the results, because the number of patients in this study with severe complications was too small (*n* = 7).

We also evaluated the efficacy of perioperative treatments such as chemotherapy, radiotherapy, and chemoradiotherapy before or after surgical resection for recurrent lesions. These therapies did not improve the OS after surgical resection for recurrent lesions. On the other hand, OS in the Yes group after surgery for recurrent lesions tended to be inferior to that in the None group (Fig. 4b). The rates of R0 resection (*P* = 0.0069) and single-organ recurrence (*P* = 0.0095) were significantly lower in the Yes group than in the None group (Online Resources 5 and 6). The low R0 resection rate may have affected the decision to perform postoperative treatment and the prognosis. Results of subgroup analyses also did not show significant changes in the prognosis, although patients who had multiple recurrent lesions or multiple-organ involvement and those with lung metastasis tended to have a favorable prognosis after undergoing perioperative chemotherapy and/or radiotherapy after recurrence. Previous reports and our study suggest that perioperative treatment might not be necessary when R0 resection is achieved for a single recurrent lesion. However, further studies are required to ascertain whether perioperative chemotherapy and/or radiotherapy should be indicated for patients with multiple recurrent lesions or multiple-organ involvement.

A limitation of this study was that it was retrospective. In addition, the present study did not compare the outcomes of surgical resection for recurrent lesions with the outcomes of other therapies such as chemotherapy, radiotherapy, or chemoradiotherapy. Therefore, we cannot decisively conclude that surgical resection for recurrent lesions is superior to other therapies. However, the overall long-term prognosis for the 186 patients in the present study was more favorable than the generally known data for the prognosis after recurrence [4, 5, 13]. The median survival time after surgical resection for recurrent lesions was 1030 days and the 5-year OS rate was 39.8% for 181 patients treated at specialized institutes in Japan. Therefore, improved prognoses may be expected after surgical resection for recurrent lesions in patients with the favorable prognostic factors identified during this study. No multi-institutional collaborative study of the resection of recurrent lesions in patients with esophageal carcinoma has included a similar number of cases as this study. Therefore, we believe that the results of the present study will provide meaningful information that can guide decision-making treatment strategies in clinical practice.

In conclusion, the results of our study suggest that surgical resection of recurrent lesions after curative esophagectomy is a useful treatment option, particularly for ESCC patients with lower pN stage, lung metastasis, long recurrence-free interval, and technically resectable recurrent lesions. When considering resection for recurrent lesions, careful attention should be focused on minimizing surgical risks as much as possible.

## Supplementary Information

Below is the link to the electronic supplementary material.

Supplementary file1 (DOCX 50 KB)

Supplementary file2 (TIF 175 KB)

Supplementary file3 (TIF 174 KB)

Supplementary file4 (TIF 280 KB)
