# Supplementary material for: Clinical outcomes of surgical resection for recurrent lesion after curative esophagectomy for esophageal squamous cell carcinoma: a nationwide, large-scale retrospective study
Source: Esophagus. 2021 Sep 12;19(1):57–68. doi: 10.1007/s10388-021-00878-2 (PMC8739466; doi:10.1007/s10388-021-00878-2)
Supplement: Supplementary file 1 — Supplementary file1 (DOCX 50 KB) [file 10388_2021_878_MOESM1_ESM.docx]

**LIST OF SUPPLEMENTAL DIGITAL CONTENT**

Online Resource 1. Details of perioperative therapy associated with prior esophagectomy.

Characteristic No. of patients

n (%)

Neoadjuvant therapy No 75 (40.3)

Chemotherapy 83 (44.6)

CRT 28 (15.1)

Regimens of neoadjuvant No 75 (40.3)

chemotherapy FP 74 (39.8)

DCF 26 (14.0)

FAP 6 (3.2)

Others 5 (2.7)

Details of neoadjuvant No 158 (84.9)

radiotherapy < 40.0Gy 14 (7.5)

40.1-50.0Gy 2 (1.1)

50.1-60.0Gy 9 (4.8)

60.1-65.4Gy 2 (1.1)

Unknown 1 (0.5)

Adjuvant therapy No 107 (57.5)

Chemotherapy 61 (32.8)

CRT 13 (7.0)

RT 3 (1.6)

Cancer vaccine 2 (1.1)

Regimens of adjuvant No 112 (60.2)

chemotherapy FP 41 (22.0)

DCF 12 (6.5)

S1 10 (5.4)

DTX 5 (2.7)

FGP 3 (1.6)

Others 3 (1.6)

Online Resource 1.

Continued

Details of adjuvant No 168 (90.3)

radiotherapy 40.1-50.0Gy 3 (1.6)

50.1-60.0Gy 14 (7.5)

Unknown 1 (0.5)

CRT: chemoradiotherapy, RT: radiotherapy, FP: 5-fluorouracil and cisplatin, DCF: docetaxel, cisplatin and 5-fluorouracil, FAP: 5-fluorouracil, doxorubicin and cisplatin, S1: tegafur, gimeracil, and oteracil potassium, DTX: docetaxel, FGP: 5-fluorouracil and nedaplatin, CDGP: nedaplatin (cis-diammine glycolato platinum)

Online Resource 2. Details of therapy before and after surgical resection for recurrent lesions

Characteristic No. of patients

n (%)

Therapy before surgery No 140 (75.3)

for recurrent lesion Chemotherapy 32 (17.2)

CRT 13 (7.0)

RT 1 (0.5)

Regimens of No 141 (76.2)

chemotherapy before FP 12 (6.5)

surgery for recurrence DCF 11 (5.9)

S1 6 (3.2)

DTX 5 (2.7)

FGP 3 (1.6)

CDGP+DTX 3 (1.6)

Others 4 (2.2)

Details of radiotherapy No 172 (92.5)

before surgery for 40.1-50.0Gy 1 (0.5)

recurrence 50.1-60.0Gy 9 (4.8)

> 60.0Gy 1 (0.5)

Unknown 3 (1.6)

Therapy after surgery No 103 (55.4)

for recurrent lesion Chemotherapy 40 (21.5)

RT 22 (11.8)

CRT 21 (11.3)

Online Resource 2.

Continued

Regimens of No 125 (67.2)

chemotherapy after FP 21 (11.3)

surgery for recurrence S1 9 (4.8)

DCF 8 (4.3)

FGP 6 (3.2)

DTX 5 (2.7)

CDGP+DTX 4 (2.2)

Others 7 (3.8)

Details of radiotherapy No 143 (76.9)

after surgery for < 40.0Gy 12 (6.5)

recurrence 40.1-50.0Gy 10 (5.4)

50.1-60.0Gy 16 (8.6)

60.1-70.0Gy 4 (2.2)

Unknown 1 (0.5)

CRT: chemoradiotherapy, RT: radiotherapy, FP: 5-fluorouracil and cisplatin, DCF: docetaxel, cisplatin and 5-fluorouracil, S1: tegafur, gimeracil, and oteracil potassium, DTX: docetaxel, FGP: 5-fluorouracil and nedaplatin, CDGP: nedaplatin (cis-diammine glycolato platinum)

Online Resource 3.

Clinicopathological characteristics of patients at initial esophagectomy according to treatment with chemotherapy and/or radiotherapy before surgical resection for recurrent lesions

Characteristic Yes None *P* value

(n = 45) (n = 136)

n (%) n (%)

Sex Male 41 (91.1) 114 (83.8) 0.3270

Female 4 (8.9) 22 (16.2)

Age 62.9 (46-76) 63.7 (40-83) 0.5708

Tumor location Cervix 0 (0.0) 9 (6.6) 0.2045

Upper thorax 6 (13.3) 25 (18.4)

Middle thorax 21 (46.7) 66 (48.5)

Lower thorax 16 (35.6) 30 (22.1)

Abdomen 2 (4.4) 6 (4.4)

cStage I 3 (6.7) 19 (14.0) 0.4076

II 16 (35.6) 42 (30.9)

III 24 (53.3) 63 (46.3)

IVa 2 (4.4) 12 (8.8)

cT T1 8 (17.8) 34 (25.0) 0.6111

T2 11 (24.4) 26 (19.1)

T3 24 (53.3) 66 (48.5)

T4 2 (4.4) 10 (7.4)

cN N0 10 (22.2) 38 (27.9) 0.5249

N1 18 (40.0) 39 (28.7)

N2 9 (20.0) 38 (27.9)

N3 6 (13.3) 13 (9.6)

N4 2 (4.4) 8 (5.9)

Online Resource 3. Continued

Comorbidities and No 29 (64.4) 81 (59.6) 0.6011

previous history Yes 16 (35.6) 55 (40.4)

Surgical procedure Subtotal esophagectomy 41 (91.1) 122 (89.7) 0.2120

Laryngopharyngoesophagectomy 0 (0.0) 7 (5.2)

Others 4 (8.9) 7 (5.2)

All postoperative No 23 (51.1) 67 (49.3) 0.8647

complications Yes 22 (48.9) 69 (50.7)

Severe complications No 37 (82.2) 108 (79.4) 0.8302

(CD grade ≥ IIIa) Yes 8 (17.8) 28 (20.6)

pStage 0 1 (2.2) 11 (8.1) 0.5746

I 5 (11.1) 21 (15.4)

II 14 (31.1) 38 (27.9)

III 21 (46.7) 58 (42.7)

IVa 4 (8.9) 8 (5.9)

pT T0 1 (2.2) 12 (8.8) 0.6578

T1 15 (33.3) 43 (31.6)

T2 5 (11.1) 16 (11.8)

T3 22 (48.9) 58 (42.7)

T4 2 (4.4) 7 (5.2)

pN N0 13 (28.9) 48 (35.3) 0.5772

N1 12 (26.7) 32 (23.5)

N2 11 (24.4) 38 (27.9)

N3 6 (13.3) 15 (11.0)

N4 3 (6.7) 3 (2.2)

Online Resource 3. Continued

Pathological effect of Grade 0 1 (2.2) 12 (8.8) 0.1612

neoadjuvant therapy Grade 1a 18 (40.0) 33 (24.3)

(n = 111) Grade 1b 3 (6.7) 9 (6.6)

Grade 2 5 (11.1) 11 (8.1)

Grade 3 1 (2.2) 11 (8.1)

Unknown 3 (6.7) 4 (2.9)

Data are presented as n (%), with the exception of age, which is presented as mean (range).

CD: Clavien-Dindo classification

Online Resource 4.

Clinical features related to surgery for recurrent lesions according to treatment with chemotherapy and/or radiotherapy before surgical resection for recurrent lesions

Characteristic Yes None *P* value

(n = 45) (n = 136)

n (%) n (%)

Number of Solitary 35 (77.8) 111 (81.6) 0.6635

recurrent lesions Multiple 10 (22.2) 25 (18.4)

Number of organs with Single 43 (95.6) 124 (91.2) 0.5226

recurrence Multiple 2 (4.4) 12 (8.8)

Residual tumor R0 39 (86.7) 113 (83.1) 0.7791

R1 2 (4.4) 10 (7.4)

R2 4 (8.9) 13 (9.6)

All postoperative No 42 (93.3) 120 (88.2) 0.3335

complications Yes 3 (6.7) 16 (11.8)

Severe complications No 45 (100.0) 129 (94.9) 0.1954

(CD grade ≥ IIIa) Yes 0 (0.0) 7 (5.1)

CD: Clavien-Dindo classification

Online Resource 5.

Clinicopathological characteristics of patients at initial esophagectomy according to treatment with chemotherapy and/or radiotherapy after surgical resection for recurrent lesions

Characteristic Yes None *P* value

(n = 79) (n = 102)

n (%) n (%)

Sex Male 69 (87.3) 86 (84.3) 0.6708

Female 10 (12.7) 16 (15.7)

Age 62.7 (46-77) 64.1 (40-83) 0.2768

Tumor location Cervix 6 (7.6) 3 (2.9) 0.5193

Upper thorax 15 (19.0) 16 (15.7)

Middle thorax 37 (46.8) 50 (49.0)

Lower thorax 17 (21.5) 29 (28.4)

Abdomen 4 (5.1) 4 (3.9)

cStage I 12 (15.2) 10 (9.8) 0.2196

II 21 (26.6) 37 (36.3)

III 42 (53.2) 45 (44.1)

IVa 4 (5.1) 10 (9.8)

cT T1 22 (27.9) 20 (19.6) 0.1224

T2 10 (12.7) 27 (26.5)

T3 41 (51.9) 49 (48.0)

T4 6 (7.6) 6 (5.9)

cN N0 22 (27.9) 26 (25.5) 0.9048

N1 24 (30.4) 33 (32.4)

N2 21 (26.6) 26 (25.5)

N3 9 (11.4) 10 (9.8)

N4 3 (3.8) 7 (6.9)

Online Resource 5. Continued

Comorbidities and No 52 (65.8) 58 (56.9) 0.2826

previous history Yes 27 (34.2) 44 (43.1)

Surgical procedure Subtotal esophagectomy 69 (87.3) 94 (92.2) 0.5587

Laryngopharyngoesophagectomy 4 (5.1) 3 (2.9)

Others 6 (7.6) 5 (4.9)

All postoperative No 40 (50.6) 50 (49.0) 0.8813

complications Yes 39 (49.4) 52 (51.0)

Severe complications No 60 (75.9) 85 (83.3) 0.2610

(CD grade ≥ IIIa) Yes 19 (24.1) 17 (16.7)

pStage 0 8 (10.1) 4 (3.9) 0.3404

I 11 (13.9) 15 (14.7)

II 19 (24.1) 33 (32.4)

III 37 (46.8) 42 (41.2)

IVa 4 (5.1) 8 (7.8)

pT T0 5 (6.3) 8 (7.8) 0.9468

T1 27 (34.2) 31 (30.4)

T2 9 (11.4) 12 (11.8)

T3 35 (44.3) 45 (44.1)

T4 3 (3.8) 6 (5.9)

pN N0 27 (34.2) 34 (33.3) 0.5095

N1 19 (24.1) 25 (24.5)

N2 25 (31.7) 24 (23.5)

N3 6 (7.6) 15 (14.7)

N4 2 (2.5) 4 (3.9)

Online Resource 5. Continued

Pathological effect of Grade 0 4 (5.1) 9 (8.8) 0.7501

neoadjuvant therapy Grade 1a 23 (29.1) 28 (27.5)

(n = 111) Grade 1b 3 (3.8) 9 (8.8)

Grade 2 7 (8.9) 9 (8.8)

Grade 3 6 (7.6) 6 (5.9)

Unknown 4 (5.1) 3 (2.9)

Data are presented as n (%), with the exception of age, which is presented as mean (range).

CD: Clavien-Dindo classification

Online Resource 6.

Clinical features related to surgery for recurrent lesions according to treatment with chemotherapy and/or radiotherapy after surgical resection for recurrent lesions

Characteristic Yes None *P* value

(n = 79) (n = 102)

n (%) n (%)

Number of Solitary 59 (74.7) 87 (85.3) 0.0884

recurrent lesions Multiple 20 (25.3) 15 (14.7)

Number of organs with Single 68 (86.1) 99 (97.1) 0.0095

recurrence Multiple 11 (13.9) 3 (2.9)

Residual tumor R0 59 (74.7) 93 (91.2) 0.0069

R1 7 (8.9) 5 (4.9)

R2 13 (16.5) 4 (3.9)

All postoperative No 70 (88.6) 92 (90.2) 0.8088

complications Yes 9 (11.4) 10 (9.8)

Severe complications No 76 (96.2) 98 (96.1) 1.0000

(CD grade ≥ IIIa) Yes 3 (3.8) 4 (3.9)

CD: Clavien-Dindo classification

Online Resource 7.

Kaplan-Meier curves of overall survival after surgical resection for recurrent lesions according to treatment with chemotherapy and/or radiotherapy (a, b) before and (c, d) after surgical resection for recurrent lesions in (a, c) patients who had solitary recurrent lesions and (b, d) patients who had multiple recurrent lesions. MST: median survival time

Online Resource 8.

Kaplan-Meier curves of overall survival after surgical resection for recurrent lesions according to treatment with chemotherapy and/or radiotherapy (a, b) before and (c, d) after surgical resection for recurrent lesions in patients with (a, c) single-organ metastasis and (b, d) multiple-organ metastases. MST: median survival time

Online Resource 9.

Kaplan-Meier curves of overall survival after surgical resection for recurrent lesions and treatment with chemotherapy and/or radiotherapy (a-c) before and (d-f) after surgical resection for recurrent lesions in patients with (a, d) lymph node recurrence, (b, e) lung recurrence, and (c, f) recurrence in other organs. LN: lymph node, MST: median survival time

Online Resource 10. The number of patients and ratio of R0 resection by each recurrent site

Characteristic n R0 R1 or R2

n (%) n (%)

Recurrent organ Lymph nodes (cervix) 65 56 (86%) 9 (14%)

Lymph nodes (thorax) 27 21 (78%) 6 (22%)

Lymph nodes (abdomen) 14 14 (100%) 0 (0%)

Lung 37 35 (95%) 2 (5%)

Brain 6 3 (50%) 3 (50%)

Skin 6 2 (33%) 4 (67%)

Liver 5 5 (100%) 0 (0%)

Adrenal gland 5 4 (80%) 1 (20%)

Kidney 4 3 (75%) 1 (25%)

Local recurrence 4 3 (75%) 1 (25%)

Others 8 6 (75%) 2 (25%)
