# Supplementary figures and images for: Clinical outcomes of surgical resection for recurrent lesion after curative esophagectomy for esophageal squamous cell carcinoma: a nationwide, large-scale retrospective study
Source: Esophagus. 2021 Sep 12;19(1):57–68. doi: 10.1007/s10388-021-00878-2 (PMC8739466; doi:10.1007/s10388-021-00878-2)

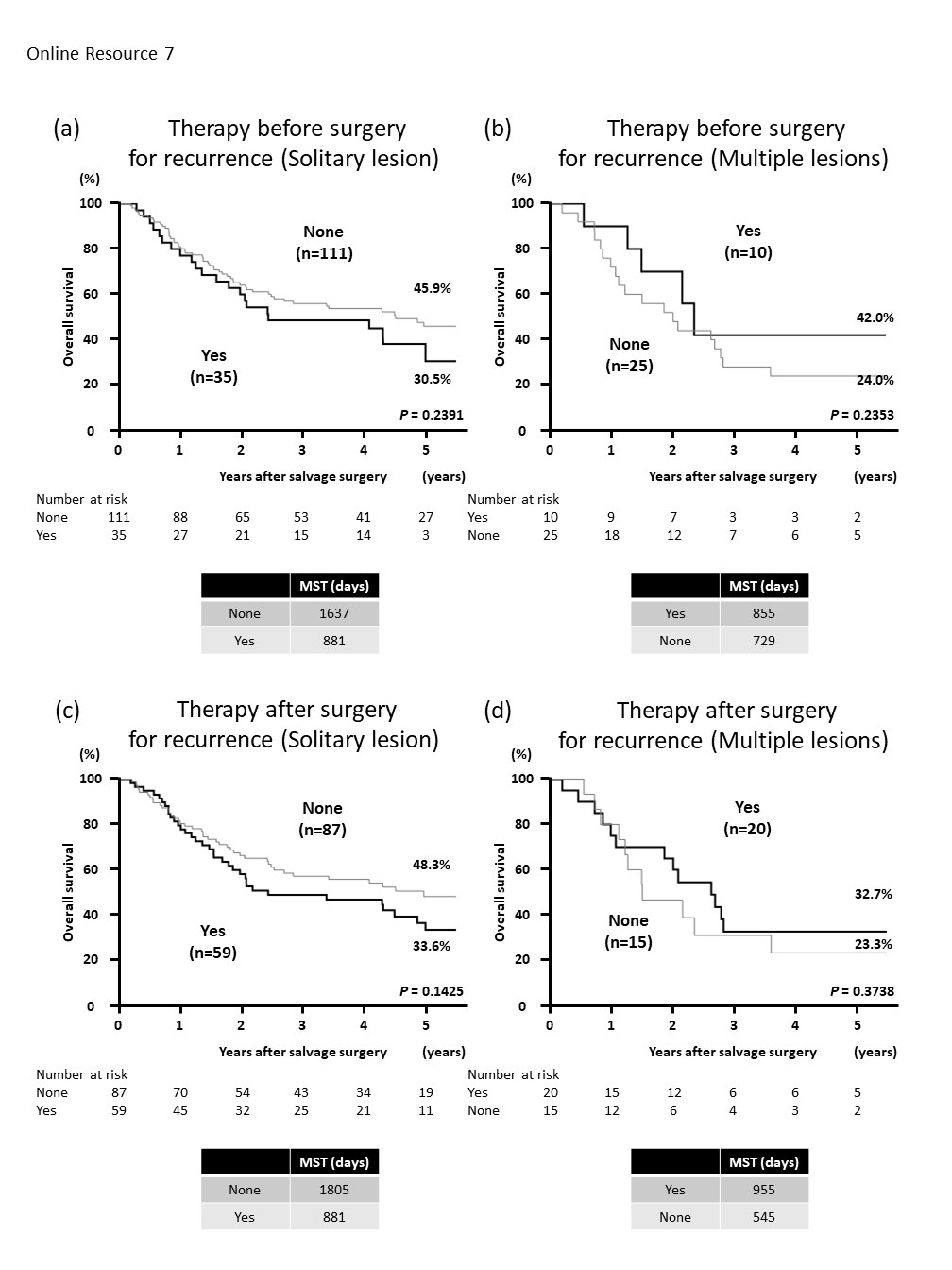

Supplement: Supplementary file 2 — Supplementary file2 (TIF 175 KB) [file 10388_2021_878_MOESM2_ESM.tif]

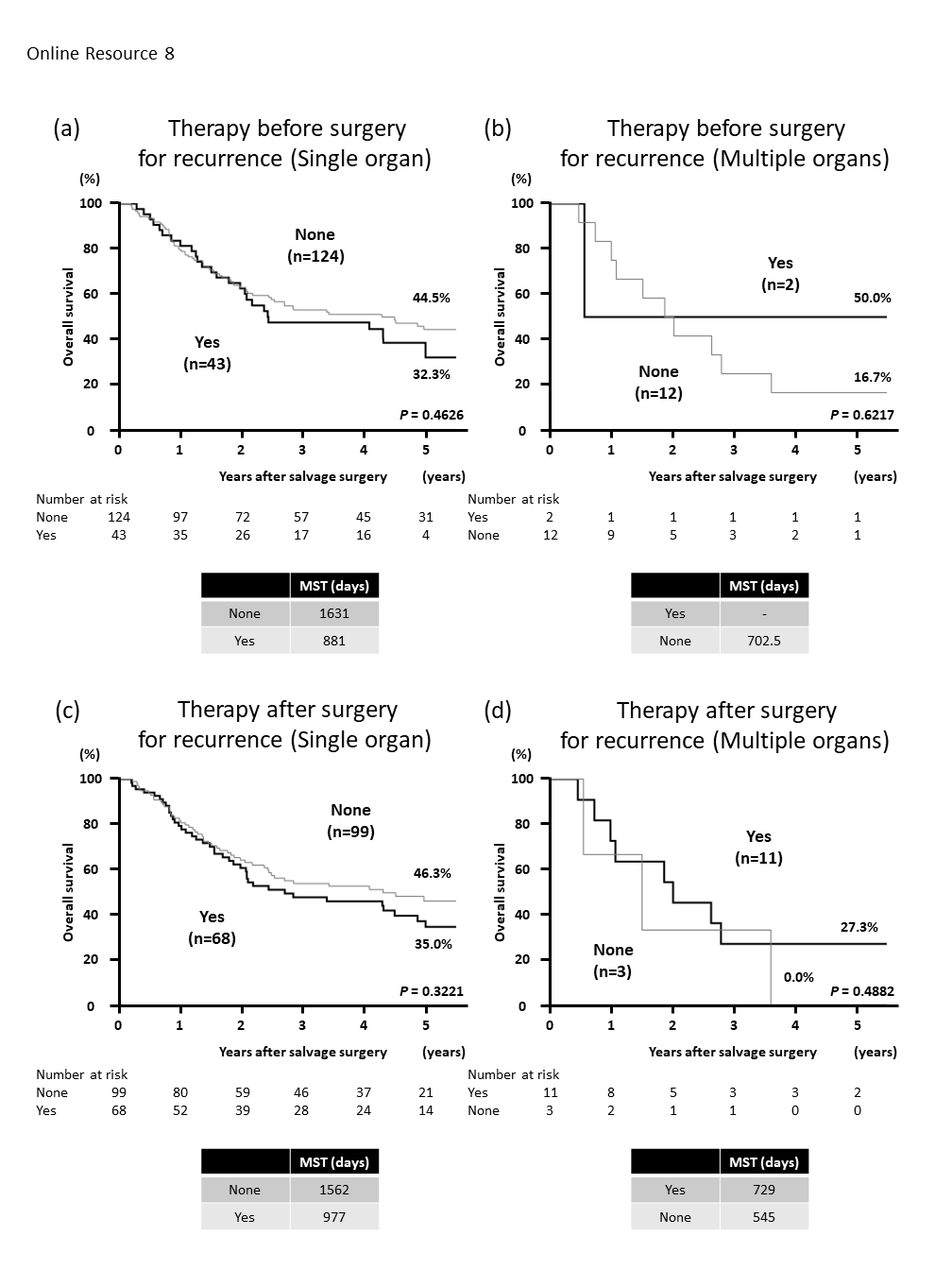

Supplement: Supplementary file 3 — Supplementary file3 (TIF 174 KB) [file 10388_2021_878_MOESM3_ESM.tif]

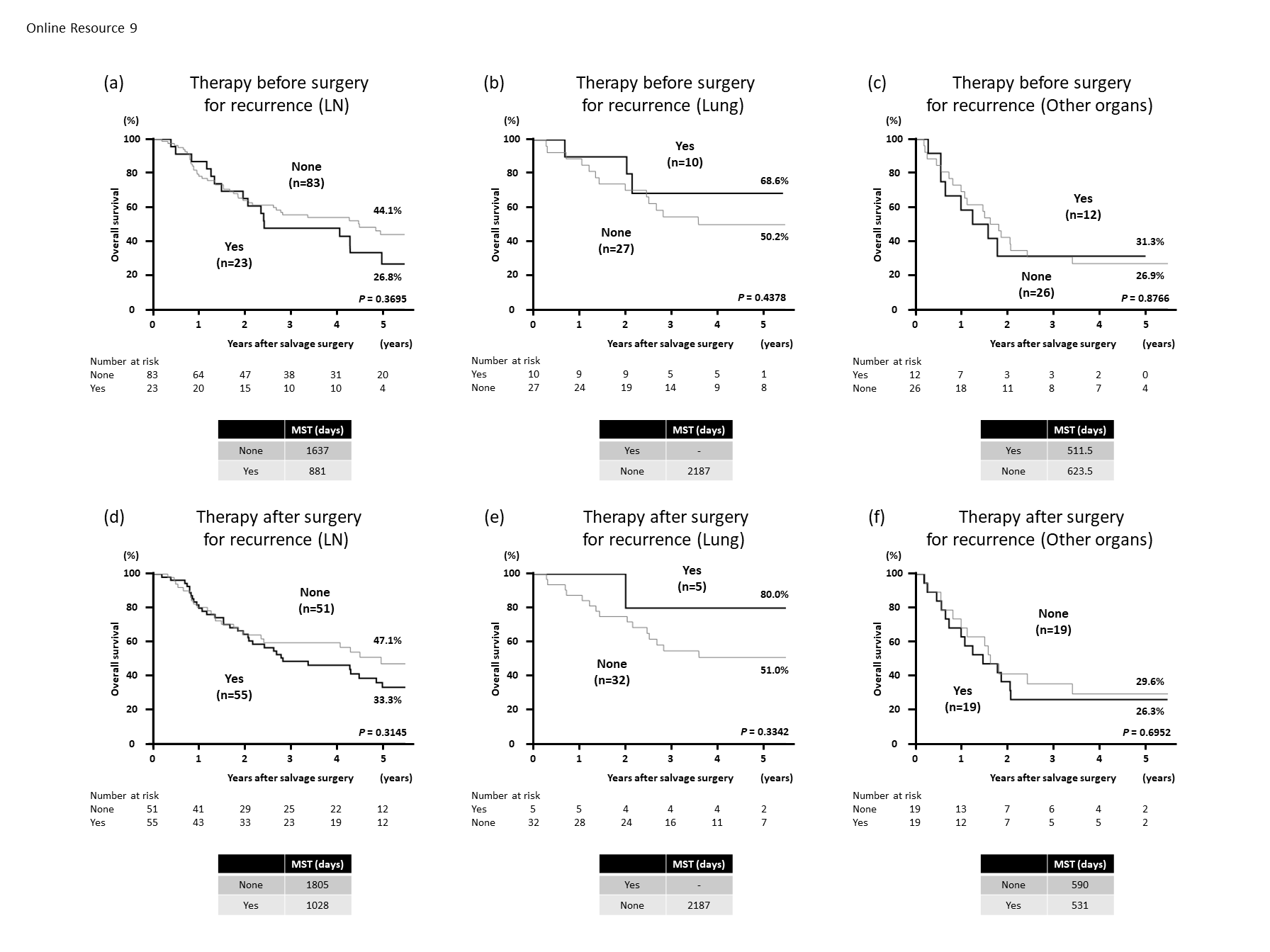

Supplement: Supplementary file 4 — Supplementary file4 (TIF 280 KB) [file 10388_2021_878_MOESM4_ESM.tif]
